# Loss of LRP1 Promotes Hepatocellular Carcinoma Progression via UFL1‐Mediated Activation of NF‐κB Signaling

**DOI:** 10.1002/advs.202401672

**Published:** 2024-10-15

**Authors:** Xingxian Guo, Fan Yang, Tianyi Liu, Amei Chen, Dina Liu, Jiangxia Pu, Can Jia, Yuanhong Wu, Junfeng Yuan, Nan Ouyang, Joachim Herz, Yinyuan Ding

**Affiliations:** ^1^ Centre for Lipid Research & Chongqing Key Laboratory of Metabolism on Lipid and Glucose Key Laboratory of Molecular Biology for Infectious Diseases (Ministry of Education) Institute for Viral Hepatitis Department of Infectious Diseases The Second Affiliated Hospital Chongqing Medical University Chongqing 400016 China; ^2^ Chongqing Key Laboratory of Translational Research for Cancer Metastasis and Individualized Treatment Chongqing University Cancer Hospital Chongqing 400030 China; ^3^ Key Laboratory of Molecular Biology for Infectious Diseases (Ministry of Education) Institute for Viral Hepatitis Department of Infectious Diseases The Second Affiliated Hospital Chongqing Medical University Chongqing 400016 China; ^4^ Department of Nephrology The First Affiliated Hospital of Chongqing Medical University Chongqing 400016 China; ^5^ Department of Molecular Genetics Department of Neuroscience Department of Neurology & Neurotherapeutics University of Texas Southwestern Medical Center Dallas TX 75390 USA

**Keywords:** hepatocellular carcinoma (HCC), low‐density lipoprotein receptor‐related protein‐1 (LRP1), NF‐κB, OGA, O‐GlcNAcylation, ubiquitin‐like modifier 1 ligating enzyme 1 (UFL1)

## Abstract

Low‐density lipoprotein receptor‐related protein‐1 (LRP1) is thought to be correlated with hepatocellular carcinoma (HCC) invasion and metastasis. However, the precise mechanism through which LRP1 contributes to HCC progression remains unclear. Here, lower LRP1 levels are associated with malignant progression, and poor prognosis in patients with HCC is shown. LRP1 knockdown enhances the tumorigenicity of HCC cells in vitro and in vivo, whereas overexpression of either LRP1 or its β‐chain has the opposite effect. Mechanistically, LRP1 knockdown promotes the binding of ubiquitin‐like modifier 1 ligating enzyme 1 (UFL1) to OGA and accelerates ubiquitin‐mediated OGA degradation, leading to increased O‐GlcNAcylation of nuclear factor‐kappa B (NF‐κB) and subsequent inhibition of pro‐apoptotic gene expression. Conversely, exogenously expressed truncated β‐chain (β∆) stabilizes OGA by disrupting the association between UFL1 and OGA, consequently abolishing the anti‐apoptotic effects of O‐GlcNAcylated NF‐κB. The findings identify LRP1, particularly its β‐chain, as a novel upstream control factor that facilitates the stabilization of the OGA protein, thereby suppressing NF‐κB signaling and attenuating HCC progression, thus suggesting a novel therapeutic strategy for HCC.

## Graphical abstract

LRP1 controls NF‐κB/Bcl‐2 signaling to affect HCC progression is shown. Hepatic LRP1 correlates with malignant progression and poor prognosis in HCC patients, and LRP1 knockdown promotes HCC cell tumorigenicity by enhancing NF‐κB O‐GlcNAcylation and thereby activating Bcl‐2 transcription, which can be reversed by overexpression of the LRP1 β‐chain, thus implicating a potential therapeutic strategy for HCC.

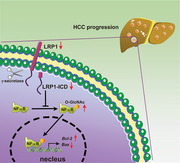

## 1 Introduction

Hepatocellular carcinoma (HCC) is a prevalent malignancy worldwide with phenotypic diversity, poor prognosis, and a high mortality rate.^[^ ^1^ ^]^ Currently, effective prevention and treatment of HCC remains a major global healthcare challenge.^[^ ^2^ ^]^ HCC exhibits a high degree of genetic heterogeneity among tumors from different patients or within tumors in the same patient, contributing to the occurrence, progression, and limited therapeutic responses of HCC.^[^ ^3^ ^]^ Therefore, a better understanding of the implications of genetic heterogeneity in HCC will yield valuable insights into the development of novel innovative therapeutic strategies against HCC.

Low‐density lipoprotein receptor‐related protein‐1 (LRP1) is a multifunctional endocytic and cell signaling receptor containing an extracellular α‐chain and a membrane‐anchored cytoplasmic β‐chain.^[^ ^4^ ^]^ In addition to its important role in lipid and glucose metabolism, LRP1 has been implicated in tumor cell proliferation, angiogenesis, invasion, and migration.^[^ ^5^ ^]^ Bian et al. demonstrated that LRP1 inhibition alleviated tumorigenesis in leukemia models by inhibiting the Notch signaling pathway.^[^ ^6^ ^]^ By contrast, reduced LRP1 expression was shown to enhance the aggressiveness and invasiveness of HCCs by increasing the expression and bioactivity of matrix metalloproteinase 9 (MMP9), which was inconsistent with the results observed in epithelial ovarian cancer cells and breast cancer.^[^ ^5^, ^7^ ^]^ Thus, the underlying mechanisms by which LRP1 is involved in HCC pathogenesis require further investigation.

Accumulating evidence has suggested that dysregulation of protein O‐linked β‐N‐acetylglucosamine (O‐GlcNAc) modification (also known as O‐GlcNAcylation) is associated with tumorigenesis.^[^ ^8^ ^]^ As a reversible post‐translational modification, O‐GlcNAcylation refers to the transfer of GlcNAc from UDP‐GlcNAc to the serine/threonine residues of proteins.^[^ ^9^ ^]^ The dynamic balance of O‐GlcNAcylation is tightly controlled by O‐GlcNAc transferase (OGT) and O‐GlcNAcase (OGA), which catalyze covalent addition and hydrolytic removal of O‐GlcNAc to and from proteins. Pharmacologically, genetically, or nutritionally, enhanced O‐GlcNAcylation was shown to promote cancer progression.^[^ ^10^ ^]^ Notably, a recent study demonstrated that high levels of glucose promoted liver tumorigenesis through advanced glycosylation end‐product‐specific receptor (AGER)‐stimulated O‐GlcNAcylation of c‐Jun, indicating a crucial role of O‐GlcNAcylation in the development of HCC.^[^ ^11^ ^]^ These results prompted us to propose dysregulation of O‐GlcNAc modification as a link connecting aberrant LRP1 expression to HCC pathogenesis.

To address this, we evaluated LRP1 expression in human HCC tumor tissues and investigated the role of LRP1 in the tumorigenicity of HCC cells in vivo and in vitro using gain‐ and loss‐of‐function methods. Our results showed that lower expression levels of hepatic LRP1 were associated with malignant progression and poor prognosis in patients with HCC. LRP1 knockdown promoted the ubiquitination and degradation of OGA, increased O‐GlcNAcylation of nuclear factor‐kappa B (NF‐κB), and inhibited the apoptosis of HCC cells, thereby enhancing the tumorigenicity of HCC cells. Conversely, overexpression of either OGA or LRP1 truncated β‐chain (β∆) ameliorated the O‐GlcNAcylation of NF‐κB and reduced the tumorigenicity of HCC cells. These findings reveal a previously unreported role of LRP1 in the regulation of OGA turnover and HCC progression, suggesting a potential new therapeutic strategy for HCC.

## 2 Results

### 2.1 Reduced LRP1 Expression Correlates with Poor Prognosis of HCC

**Figure 1:**
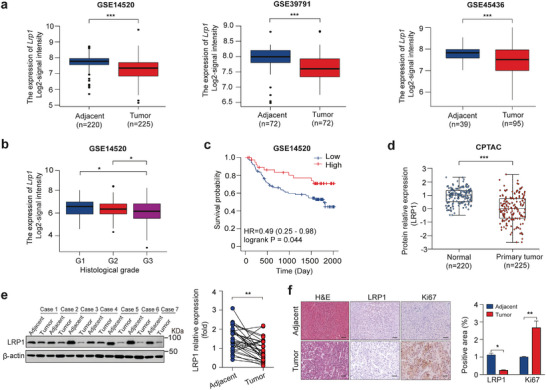
Lower LRP1 expression is associated with poor prognosis of HCC. a) Comparison of *Lrp1* mRNA levels in HCC and noncancerous liver tissues in the GSE14520, GSE39791, and GSE45436 datasets. b) Correlation between *Lrp1* mRNA levels and clinical histological grades in the GSE14520 cohort. c) Analysis of Kaplan‐Meier survival curves for patients with HCC in the GSE14520 cohort. d) Comparison of LRP1 protein levels between tumor and normal groups in the CPTAC dataset. e) Representative western blots and analysis of LRP1 protein levels in HCC and matched non‐tumor tissues from 30 patients. f) Representative H&E staining and IHC images and quantification of LRP1 and Ki67 in HCC or adjacent tissues (*n* = 5 subjects per group). Scale bar, 50 µm. Data are mean ± SEM. Statistical analyses were performed using unpaired 2‐tailed Student's t‐test (a, d, f), one‐way ANOVA with Tukey's multiple comparison tests (b), log‐rank [Mantel‐Cox] test (c), or two‐tailed paired t‐test (e). *p < 0.05, **p < 0.01, ***p < 0.001.

Bioinformatics analysis showed that *Lrp1* transcription levels in HCC tissues were remarkably decreased compared to those in adjacent non‐tumorous tissues in three GEO datasets, GSE14520, GSE39791, and GSE45436 (**Figure** **1**a). Subgroup analysis revealed that HCC patients with a lower mRNA level of *Lrp1* tended to have a higher histological grade and worse prognosis than those with a high *Lrp1* level in the GSE14520 dataset (Figure 1b,c). Similarly, we found that LRP1 protein levels in HCC tissues were significantly lower than those in normal liver tissues in the Clinical Proteomic Tumor Analysis Consortium (CPTAC) dataset (Figure 1d). Moreover, immunoblot and immunohistochemistry (IHC) staining revealed that LRP1 and Ki67 levels were clearly decreased and increased, respectively, in HCC tissues compared to those in paired adjacent non‐tumor tissues in 30 HCC cases (Figure 1e,f). Taken together, these results suggest that LRP1 expression is downregulated in HCC tissues, which may contribute to malignant progression and poor prognosis of patients with HCC.

### 2.2 Modulation of LRP1 Expression Affects Tumorigenicity of HCC Cells

**Figure 2:**
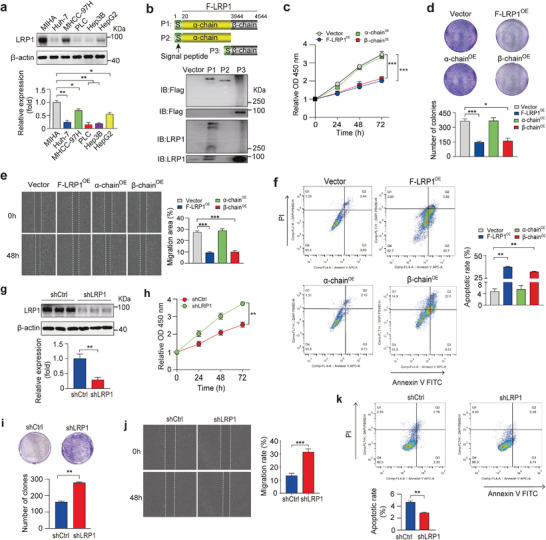
Overexpression and knockdown of LRP1 impact the proliferation and migration of HCC cells. a) LRP1 expression in normal hepatocytes and hepatoma cell lines. b) Schematic illustrating the generation of constructs carrying full‐length LRP1 (F‐LRP1) or its α‐ or β‐chain coding sequences, and western blot analysis of Flag‐tagged protein and LRP1 expression using antibodies against Flag or the C‐terminal fragment of LRP1 in PLC5 cells expressing F‐LRP1 (P1), α‐ (P2), and β‐chain (P3), respectively. c–d) CCK‐8 and colony formation assays in PLC5 cells transfected with the indicated plasmids. e) Wound healing assay in MHCC‐97H cells at 48 h after the indicated treatment. f) FACS analysis with Annexin V/PI staining in MHCC‐97H cells under the indicated treatment. g) Representative western blots for LRP1 expression in MHCC‐97H cells infected with lentivirus carrying a nontarget control (shCtrl) or shRNA targeting the human *Lrp1* gene (shLRP1) and quantification of three independent experiments. h‐k) The CCK8 (h), colony formation (i), wound healing assay (j), and FACS analysis (k) in shCtrl and shLRP1 MHCC‐97H cells. Data are mean ± SEM. *n* = 3 independent experiments for panels a, e, f, j, and k; *n* = 5 independent experiments for panels c, d, h, and i. Statistical analyses were performed using one‐way ANOVA with Tukey's multiple comparison tests (a, d, e, f), two‐way ANOVA with Tukey's or Sidak's post‐hoc multiple comparison tests (c, h), or unpaired 2‐tailed Student's t‐test (g, i, j, k). *p < 0.05, **p < 0.01, ***p < 0.001.

To elucidate the role of LRP1 in HCC tumorigenesis, we first investigated LRP1 expression in a normal hepatocyte line, MIHA, and several HCC cell lines. Our findings showed that LRP1 protein levels in HCC cell lines were significantly lower than those in MIHA cells (**Figure** **2**a). We then generated plasmids containing full‐length (F‐LRP1) or truncated human LRP1 coding sequences and examined the effects of LRP1 and its α‐ or β‐chain overexpression on the proliferation and migration abilities of PLC5 and Huh‐7 cells, two of HCC cell lines with lower LRP1 expression (Figure 2b; Figure S1a, Supporting Information). Cell Counting Kit‐8 (CCK‐8), colony formation, and scratch wound healing assays indicated that overexpression of LRP1 or its β‐chain, rather than the α‐chain, markedly suppressed the proliferation and migration of PLC5 and Huh‐7 cells, accompanied by enhanced apoptosis, implicating a fundamental role for the LRP1 β‐chain in the HCC process (Figure 2c–f; Figure S1b–e, Supporting Information). We also used lentivirus‐mediated RNA interference to knock down LRP1 expression in HCC cell lines with higher LRP1 levels (Figure 2g; Figure S2a, Supporting Information). LRP1 knockdown significantly enhanced the proliferation of both MHCC‐97H and HepG2 cells (Figure 2h,i; Figure S2b,c, Supporting Information). Moreover, LRP1‐deleted cells exhibited a higher migratory potential in scratch wound healing assays (Figure 2j; Figure S2d, Supporting Information). Flow cytometry analysis further revealed that LRP1 knockdown increased the percentage of apoptotic MHCC‐97H cells (Figure 2k). To exclude any non‐specific effects of shRNA expression, we treated HepG2 cells with another lentiviral shRNA targeting different 3′‐UTR regions of the LRP1 transcript and observed similar results (Figure S2a–e, Supporting Information). Collectively, these results demonstrate that LRP1 overexpression and knockdown have opposite effects on the tumorigenic potential of HCC cells.

### 2.3 LRP1 Regulates the Tumorigenicity of HCC Cells via Manipulation of NF‐κB O‐GlcNAcylation

**Figure 3:**
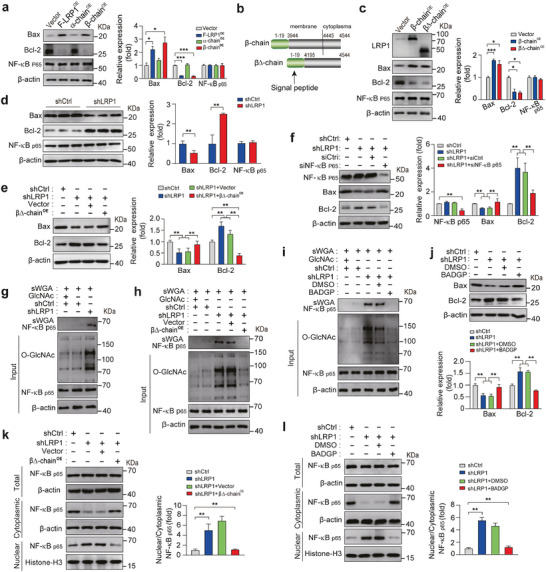
LRP1 affects the expression of apoptosis‐regulating proteins by modifying O‐GlcNAcylation of NF‐κB. a) The expression of Bax, Bcl‐2, and NF‐κB p65 in MHCC‐97H cells overexpressing F‐LRP1 and its α‐ or β‐chain. b) Schematic showing the generation of constructs carrying β‐chain or truncated β‐chain (β∆‐chain) coding sequences. c) Expression of Bax, Bcl‐2, and NF‐κB p65 in MHCC‐97H cells overexpressing β‐chain or β∆‐chain. d) Expression of Bax, Bcl‐2, and NF‐κB p65 in MHCC‐97H cells with and without LRP1 knockdown. e) Expression of Bax and Bcl‐2 in shCtrl and shLRP1 MHCC‐97H cells transfected with or without the β∆‐chain. f) Expression of Bax and Bcl‐2 in LRP1‐knockdown MHCC‐97H cells treated with control siRNA (siCtrl) or siRNA targeting the human *Nfκb p65* gene (siNF‐κB p65). g) Analysis of NF‐κB p65 O‐GlcNAcylation in MHCC‐97H cells infected with shCtrl or shLRP1 lentivirus using sWGA pull‐down assays. The monosaccharide inhibitor GlcNAc (20 mM) was used as a negative control in the sWGA‐lectin‐affinity purification process. h) Detection of NF‐κB p65 O‐GlcNAcylation in shCtrl and shLRP1 MHCC‐97H cells transfected with or without the β∆‐chain. i–j) Assessment of NF‐κB p65 O‐GlcNAcylation (i), Bax, and Bcl‐2 expression (j) in shCtrl and shLRP1 MHCC‐97H cells treated with or without 5 mM BADGP for 24 h. k) Cytoplasmic and nuclear NF‐κB P65 levels in shCtrl and shLRP1 MHCC‐97H cells overexpressing with or without the β∆‐chain. l) Cytoplasmic and nuclear NF‐κB P65 levels in shCtrl and shLRP1 MHCC‐97H cells treated with or without 5 mM BADGP for 24 h. Values are presented as the mean ± SEM. *n* = 3 independent experiments for panels a, c‐f, j–l. Statistical analyses were performed using one‐way ANOVA with Tukey's multiple comparison tests (a, c, e, f, j–l) or unpaired 2‐tailed Student's t‐test (d). * *p* < 0.05, **p < 0.01, ***p < 0.001.

We then investigated the expression of apoptosis‐regulating proteins in HCC cells and found that PLC5 cells overexpressing F‐LRP1 and its β‐, but not α‐ chain, led to increased Bax expression and decreased Bcl‐2 expression (**Figure** **3**a). To gain insight into the mechanism by which the LRP1 β‐chain modulates apoptosis of HCC cells, we constructed a plasmid carrying a β∆‐chain coding sequence and observed similar changes in Bax and Bcl‐2 expression in β∆‐chain‐overexpressing cells (Figure 3b,c). By contrast, LRP1 knockdown exerted opposite effects in both MHCC‐97H and HepG2 cells, which were reversed by β∆‐chain overexpression (Figure 3d,e; Figure S2e, Supporting Information). Surprisingly, neither knockdown nor overexpression of F‐LRP1 or β‐chain disturbed the levels of total cellular nuclear factor‐kappa B (NF‐κB), a key upstream transcription factor of apoptosis‐related genes (Figure 3a,c,d). However, silencing of NF‐κB abolished the effects of LRP1 knockdown on the expression of Bcl‐2 and Bax, suggesting that an NF‐κB‐related mechanism is involved in LRP1 knockdown‐mediated attenuation of apoptosis (Figure 3f). Previous studies demonstrated that cellular O‐GlcNAcylation affected the nuclear translocation of NF‐κB p65 in the progression of cholangiocarcinoma, leading to the activation of transcriptional activity.^[^ ^12^ ^]^ Then we tested whether NF‐κB p65 is modified by O‐GlcNAcylation in the absence of LRP1 using succinylated wheat germ agglutinin (sWGA) pull‐down assay to specifically identify O‐GlcNAcylated proteins. sWGA experiments showed that O‐GlcNAcylation of NF‐κB p65 was markedly enhanced along with increased cellular O‐GlcNAcylation levels in LRP1‐deficient cells (Figure 3g). Moreover, both β∆‐chain overexpression and administration of BADGP, an inhibitor of OGT, restored cellular O‐GlcNAcylation and O‐GlcNAcylated NF‐κB p65 levels to closely match those of the control group and recapitulated the effects of NF‐κB p65 silencing on Bax and Bcl‐2 expression in LRP1‐deleted cells (Figure 3e,h–j). Subcellular distribution analysis by immunoblotting and immunofluorescent staining showed a preferential nuclear retention pattern of NF‐κB p65 in LRP1‐knockdown cells, which was markedly reversed by β∆‐chain overexpression and BADGP administration (Figure 3k,l; Figure S3a–c, Supporting Information), suggesting that NF‐κB p65 O‐GlcNAcylation affects its subcellular localization. Taken together, these data show that LRP1 regulates HCC cell apoptosis through modulation of NF‐κB O‐GlcNAcylation and localization.

### 2.4 LRP1 Knockdown Elevates Cellular O‐GlcNAcylation Levels Through Upregulation of Ubiquitin‐Mediated OGA Degradation

**Figure 4:**
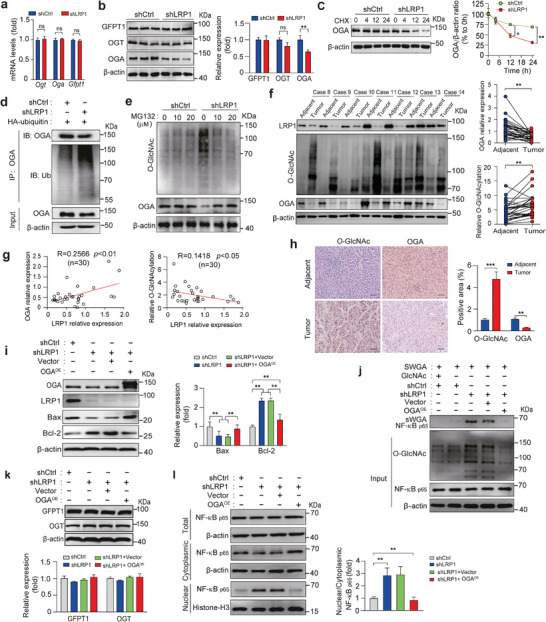
LRP1 deficiency facilitates ubiquitin‐mediated degradation of OGA protein. a) RT‐PCR analysis of *Ogt*, *Oga*, and *Gfpt1* mRNA levels in shCtrl and shLRP1 MHCC‐97H cells. b) Expression of GFPT1, OGA, and OGT in shCtrl and shLRP1 MHCC‐97H cells. c) Analysis of OGA protein stability in shCtrl and shLRP1 cells using CHX chase assay. d) Detection of ubiquitination of endogenous OGA by immunoprecipitation with an anti‐ubiquitin antibody and immunoblotting with the indicated antibodies in shCtrl and shLRP1 cells expressing HA‐ubiquitin. e) Immunoblotting analysis of OGA protein and overall O‐glycosylation expression in shCtrl and shLRP1 cells pretreated with or without MG‐132 at the indicated concentration for 24 h. f) Representative western blots of LRP1, O‐GlcNAc, and OGA proteins and quantification of OGA and O‐GlcNAcylation levels in HCC and matched non‐tumor tissues from 30 patients. g) Correlation analysis of OGA expression and overall O‐GlcNAcylation levels with LRP1 expression levels in HCC and adjacent normal tissues from 30 patients. h) Representative H&E staining and IHC images, and quantification of O‐GlcNAc and OGA staining in HCC or adjacent tissues (*n* = 5 subjects per group). Scale bar, 50 µm. i) Representative western blots and quantification of OGA, LRP1, Bax, and Bcl‐2 expression in MHCC‐97H cells under the indicated treatment. j‐k) Determination of NF‐κB p65 O‐GlcNAcylation (j), and GFPT1 and OGT expression (k) in shCtrl and shLRP1 cells overexpressing OGA. l) Cytoplasmic and nuclear NF‐κB P65 levels in shCtrl and shLRP1 cells overexpressing OGA. Data are mean ± SEM. *n* = 3 independent experiments for panels a, c, i, and k‐l. n = 30 subjects for panels f‐g. Statistical analyses were performed using unpaired 2‐tailed Student's t‐test (a, b, h), two‐way ANOVA with Sidak's post‐hoc multiple comparison tests (c), two‐tailed paired t‐test (f), Pearson's correlation test (g), or one‐way ANOVA with Tukey's multiple comparison tests (i, k–l). *p < 0.05, **p < 0.01, ***p < 0.001. ns indicates no significant differences.

These results raise the question of how LRP1 deficiency increases the cellular O‐GlcNAcylation levels. Generally, cellular O‐GlcNAcylation levels are dependent on the concerted actions of GFPT1, OGT, and OGA. As shown in **Figure** **4**a, the mRNA levels of *Oga*, *Ogt*, and *Gfpt1* did not change between the shCtrl and shLRP1 groups. However, OGA protein levels were significantly decreased in response to LRP1 silencing, whereas the expression levels of GFPT1 and OGT remained unaltered (Figure 4b). CHX‐chase experiments further showed that OGA levels were degraded much faster in shLRP1 cells in parallel with increased ubiquitination of OGA protein, whereas LRP1 deletion‐induced reduction in OGA expression and elevation of global O‐GlcNAcylation levels were completely reversed in the presence of MG132 (Figure 4c–e). Moreover, the correlation among LRP1 expression, total O‐GlcNAcylation levels, and OGA expression was validated in the liver tissues of patients with HCC. Both immunoblot and IHC staining revealed that LRP1 levels were clearly decreased in HCC tissues, accompanied by increased O‐GlcNAcylation levels and reduced OGA levels when compared with those in paired adjacent non‐tumor tissues (Figure 4f–h). Next, we overexpressed OGA to elucidate its role in LRP1‐deleted MHCC‐97H cells. In response to OGA overexpression, the enhanced proliferation and migration capabilities of MHCC‐97H cells caused by LRP1 silencing were markedly attenuated, concomitant with a visible recovery of apoptotic rates and protein expression of Bax and Bcl‐2 (Figure 4i; Figure S4a–d, Supporting Information). Moreover, upregulation of total O‐GlcNAcylation levels and NF‐κB p65 O‐GlcNAcylation in LRP1‐knockdown cells was blocked by OGA overexpression, with no change in the expression of GFPT1 and OGT (Figure 4j,k). Consistently, OGA overexpression promoted nuclear exclusion of NF‐κB p65 in shLRP1 MHCC‐97H cells (Figure 4l; Figure S4e, Supporting Information). Collectively, these results suggest that LRP1 deficiency drives tumor‐promoting activities in HCC cells by accelerating ubiquitin‐dependent degradation of OGA.

### 2.5 LRP1 β‐Chain Reverses Malignant Phenotypes of HCC In Vitro and In Vivo

**Figure 5:**
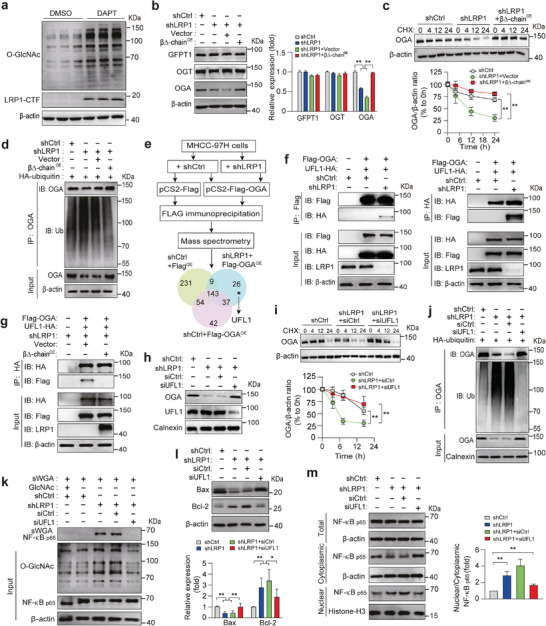
LRP1 β‐chain overexpression reverses the increased O‐GlcNAcylation and decreased OGA expression in LRP1‐knockdown cells. a) Overall O‐GlcNAcylation levels in MHCC‐97H cells treated with or without 1 mM DAPT for 24 h. b) Representative western blots and quantification of GFPT1, OGT, and OGA expression in MHCC‐97H cells under the indicated treatment conditions. c) Evaluation of OGA protein stability by CHX chase assay in HCC cells under the indicated treatment. d) Immunoblots for the detection of OGA and ubiquitin levels in cell lysates immunoprecipitated with ubiquitin. e) An experimental approach to identify proteins associated with OGA in shCtrl and shLRP1 MHCC‐97H cells. f) Immunoblot analysis of reciprocal Co‐IP of Flag‐OGA, followed by western blot analysis of Flag‐OGA and UFL1‐HA, or vice versa, from lysates of MHCC‐97H cells overexpressing Flag‐tagged OGA and HA‐tagged UFL1, in combination with and without LRP1 silencing. g) Reciprocal IP analysis to detect the association between Flag‐OGA and UFL1‐HA in cell lysates from LRP1‐deficient cells overexpressing Flag‐tagged OGA and HA‐tagged UFL1 in combination with and without LRP1 β∆‐chain expression. h) Immunoblotting analysis of OGA and UFL1 expression in shCtrl and shLRP1 cells transfected with siUFL1 or siCtrl. i) Analysis of OGA protein stability by CHX chase assay in HCC cells under the indicated treatment. j) Representative western blots for OGA and ubiquitinated OGA levels in shCtrl‐ and shLRP1‐lentivirus infected MHCC‐97H cells at various combinations as indicated. k‐m) Detection of NF‐κB p65 O‐GlcNAcylation (k), Bax and Bcl‐2 expression (l), and cytoplasmic and nuclear NF‐κB P65 levels (m) in shCtrl‐ and shLRP1 MHCC‐97H cells transfected with siCtrl or siUFL1. Data are mean ± SEM. *n* = 3 independent experiments (b–c, i, l–m). Statistical analyses were performed using one‐way ANOVA with Tukey's multiple comparison tests (b, l‐m), or two‐way ANOVA with Tukey's multiple comparison tests (c, i). *p < 0.05, **p < 0.01.

Considering that γ‐secretase‐mediated cleavage of the C‐terminal fragment (CTF) of the LRP1 β‐chain liberates the intracellular domain (ICD) and thereby regulates gene transcription and protein expression, we surmised that LRP1 might modify OGA degradation and consequently affect O‐GlcNAc modification through the LRP1‐ICD.^[^ ^13^ ^]^ As shown in **Figure** **5**a, the overall O‐GlcNAcylation levels were significantly increased along with the accumulation of LRP1‐CTF after exposure of MHCC‐97H cells to DAPT, a γ‐secretase inhibitor. Consistently, overexpression of the LRP1 β∆‐chain abrogated the increase in overall O‐GlcNAcylation levels and decrease in OGA expression caused by LRP1 knockdown (Figure 5b). Accordingly, we observed that the subsequent turnover and ubiquitination of OGA returned to normal levels in LRP1‐deficient cells expressing the LRP1 β∆‐chain (Figure 5c,d). To further explore the mechanism underlying the effects of LRP1 on the ubiquitination of OGA, Flag‐tagged OGA interactomes in MHCC‐97H cells, with and without LRP1 knockdown, were screened using a tandem affinity approach followed by mass spectrometry (Figure 5e). We identified a potential OGA‐interacting protein in shLRP1 cells, ubiquitin‐like modifier 1 ligating enzyme 1 (UFL1), which is the only E3 ligase known to mediate UFMylation and is highly expressed in the liver.^[^ ^14^ ^]^ Reciprocal immunoprecipitation and immunofluorescent staining revealed a direct association between OGA and UFL1 in the absence of LRP1 (Figure 5f; Figure S5, Supporting Information). We found that this association was completely abolished in shLRP1 cells expressing the LRP1 β∆‐chain (Figure 5g). To confirm the role of UFL1 in OGA ubiquitylation and degradation, we used siRNA to knock down UFL1 expression and found that the reduction in OGA expression in LRP1‐deficient MHCC‐97H cells was eliminated after treatment with UFL1‐siRNA (Figure 5h), accompanied by an obvious increase in OGA stability and a significant decrease in ubiquitylated‐OGA levels (Figure 5i,j). Moreover, UFL1 silencing led to the recovery of NF‐κB p65 O‐GlcNAcylation and apoptosis‐related protein expression as well as tumorigenicity in LRP1‐knockdown MHCC‐97H cells (Figure 5k,l; Figure S6a–d, Supporting Information). Meanwhile, UFL1 silencing attenuated the effect of LRP1 knockdown on nuclear retention of NF‐κB p65 (Figure 5m; Figure S6e, Supporting Information). Collectively, these observations suggest that the LRP1 β‐chain maintains OGA stability by disrupting the association between UFL1 and OGA.

**Figure 6:**
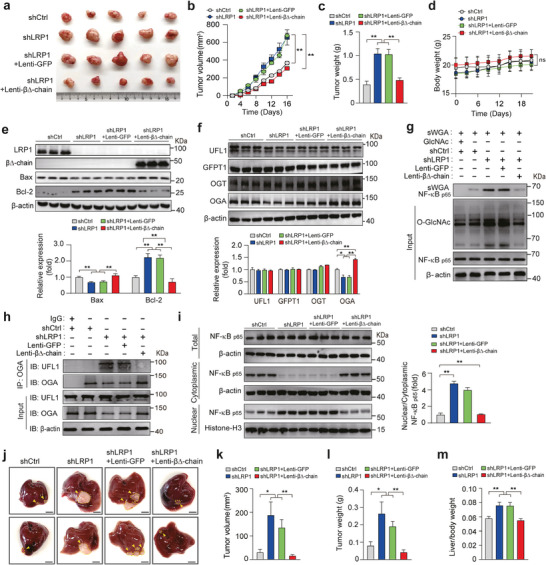
LRP1 β∆‐chain overexpression reverses the effects of LRP1 deletion on the progression of hepatoma xenograft tumors. a) Photographs of subcutaneous xenografts. b–d) Comparison of tumor volume (b), weight (c), and ponderal growth (d) among the four groups under the indicated treatment (*n* = 5 mice per group). e) Western blot analysis of LRP1, β∆‐chain, Bax, and Bcl‐2 expression in xenograft tumors. f) Western blot analysis of GFPT1, OGA, and OGT expression levels. g) Detection of NF‐κB p65 O‐GlcNAcylation in xenograft tumors. h) Reciprocal IP analysis to detect the association between OGA and UFL1 in xenograft tumors. i) Immunoblotting analysis of NF‐κB p65 in nuclear and cytosolic fractions isolated from subcutaneous xenograft tumors. j) Respective images of the orthotopic liver tumors derived from MHCC‐97H cells in nude mice (Scale bar, 5 mm). k‐m) Comparison of orthotopic tumor volume (k), weight (l), and the ratio of liver weight to body weight (m) among the four groups of nude mice (*n* = 5–6 mice per group). Data are mean ± SEM. Statistical analyses were performed using two‐way ANOVA with Tukey's multiple comparison tests (b, d) or one‐way ANOVA with Tukey's multiple comparison tests (c, e–f, i, k–m). *p < 0.05, **p < 0.01. ns indicates no significant differences.

We next examined the effects of LRP1 β‐chain overexpression on HCC pathogenesis in vitro and found that LRP1 β∆‐chain overexpression inhibited HCC cell growth and migration, and induced apoptosis in LRP1‐knockdown cells (Figures S7a–d and **S8**a–d, Supporting Information). Finally, we evaluated the in vivo effects of LRP1 expression in hepatoma xenografts. The nude mice were subcutaneously transplanted with shCtrl and shLRP1 MHCC‐97H cells overexpressing with or without LRP1 β∆‐chain, and xenograft growth was monitored by measuring tumor volume. As shown in **Figure** **6**a–d, LRP1 knockdown resulted in large‐sized subcutaneous xenografts, concomitant with a significantly higher tumor growth rate and weight, despite no difference in ponderal growth compared to the control conditions. Conversely, the increased tumorigenic capacity in subcutaneous xenografts with LRP1 knockdown was substantially reversed by lentivirus‐mediated overexpression of the LRP1 β∆‐chain, leading to a decrease in the tumor growth rate and tumor weight to a level comparable to that in the control group. Subsequent immunoblot analysis of resected tumor tissue showed that the LRP1 β∆‐chain exerted pro‐apoptotic effects by upregulating Bax and downregulating Bcl‐2 expression (Figure 6e). OGA expression and NF‐κB p65 O‐GlcNAcylation were restored, while the expression of GFPT1 and OGT did not change (Figure 6f,g). Consistently, reciprocal immunoprecipitation revealed a direct association between OGA and UFL1 in the absence of LRP1, and this association was completely abolished by overexpression of the LRP1 β∆‐chain (Figure 6h). Moreover, LRP1 β∆‐chain decreased nuclear retention of NF‐κB p65 in shLRP1 group (Figure 6i). Similar results were also observed in an orthotopic xenograft model of HCC. The tumor volume, weight, and ratio of liver weight to body weight in nude mice with orthotopic liver implantation of LRP1 knockdown MHCC‐97H cells were significantly increased compared to those in control mice at the end of the experiment (Figure 6j–m). Notably, stable expression of the β∆‐chain significantly reduced the tumor burden in nude mice bearing LRP1‐deficient cells, decreasing the tumor volume and weight to a level lower than that in mice bearing control cells, although the difference was not significant. Taken together, our results indicate that LRP1 β∆‐chain overexpression suppresses HCC tumorigenesis by reducing OGA turnover, both in vivo and in vitro.

## 3 Discussion

LRP1 expression is dysregulated in multiple tumors, including prostate, breast, colon, thyroid, and endometrial cancers, implicating a functional role for LRP1 in cancer susceptibility and progression.^[^ ^7^, ^15^ ^]^ Recently, LRP1 was reported to interact with apoE to promote colorectal cancer (CRC) migration and invasion.^[^ ^16^ ^]^ However, Rong et al. demonstrated that LRP1 mediated the inhibitory effects of fucosyltransferase 2 (FUT2) on CRC metastasis.^[^ ^17^ ^]^ Therefore, the emerging controversy regarding LRP1 function in oncogenesis encourages further studies to elucidate the precise mechanisms through which LRP1 influences HCC emergence and metastasis. In the present study, we found that LRP1 was frequently under‐expressed in both human HCC tissues and proliferating HCC cell lines, which was closely associated with high‐grade HCC and poor prognosis in HCC patients, in contrast to its effect on glioblastoma.^[^ ^5d^ ^]^ Moreover, LRP1 deficiency enhanced HCC cell proliferation, migration, and survival, thus promoting HCC cell xenograft tumor formation. By contrast, overexpression of F‐LRP1 had opposite effects on the tumorigenicity of HCC cells. We then adopted a truncation strategy to explore the structural basis and demonstrated that overexpression of both β‐chain and truncated β∆‐chain, rather than the extracellular (α) chain of LRP1, recapitulated the inhibitory effects of F‐LRP1. These results together further strengthen the anti‐tumor role of LRP1 in HCC progression.

Although one study showed that a low level of LRP1 expression enhanced the aggressiveness and invasiveness of SMCC‐7721 cells by upregulating the bioactivity and expression of MMP9,^[^ ^7b^ ^]^ this remains controversial, as other studies have concluded that LRP1 contributes to the migration and invasion of epithelial ovarian cancer cells by inducing MMP9 expression.^[^ ^5^, ^18^ ^]^ In addition, LRP1 silencing was reported to prevent MMP9 activation in hypoxic human vascular smooth muscle cells.^[^ ^19^ ^]^ However, we did not observe any changes in MMP9 expression caused by LRP1 knockdown or β∆‐chain overexpression in HCC cells (Figure S9, Supporting Information). Instead, we found obvious activation of the NF‐κB/Bcl‐2 anti‐apoptotic signaling pathway in parallel with enhanced tumorigenicity in LRP1‐knockdown HCC cells, which was attributed to increased O‐GlcNAcylation of NF‐κB p65 and the consequent upregulation of its anti‐apoptotic activity. It is well documented that NF‐κB transcriptional activity is tightly controlled by three inhibitors of NF‐κB (IκBs). Emerging evidence suggests that p65, one of five NF‐κB DNA‐binding proteins, is subjected to post‐translational modifications such as phosphorylation, acetylation, and O‐GlcNAcylation, thereby modulating NF‐κB functions in inflammation, cell proliferation, differentiation, and apoptosis.^[^ ^12^, ^20^ ^]^ NF‐κB p65 phosphorylation at Ser‐276 or Ser‐536 promotes its binding with the co‐activator CBP/P300, thereby enhancing NF‐κB gene transcription.^[^ ^21^ ^]^ Interestingly, Ma et al. further revealed that elevation of O‐GlcNAcylation stimulates the expression of the upstream kinases IKKα and IKKβ, consequently leading to the phosphorylation of NF‐κB p65 at Ser‐536 and its activation.^[^ ^22^ ^]^ Previous studies have proposed that macrophage LRP1 inhibits NF‐κB activity through a ligand‐specific mechanism or downregulation of cell‐surface tumor necrosis factor receptor‐1 (TNFR1).^[^ ^23^ ^]^ In the current study, both the β‐chain and truncated β∆‐chain completely mimicked the anti‐tumor effects of F‐LRP1 and corrected the aberrant expression of apoptosis‐related genes through diminishment of NF‐κB p65 O‐GlcNAcylation, strongly implicating that the ligand‐binding mechanism was not responsible for the tumor‐preventing role of LRP1.

It is well documented that aberrant O‐GlcNAcylation leads to metabolic reprogramming, fueling cancer malignancies and promoting tumor progression.^[^ ^24^ ^]^ OGA is the sole enzyme responsible for the removal of GlcNAc moieties from proteins and thus plays an important role in O‐GlcNAcylation and cancer metabolism.^[^ ^25^ ^]^ Consistent with this, our results revealed that LRP1 deficiency was correlated with reduced OGA expression, concomitant with increased global O‐GlcNAcylation, O‐GlcNAcylated NF‐κB p65 levels, and nuclear NF‐κB p65 distribution in human HCCs. Overexpression of both OGA and β∆‐chain reversed the increase in cellular O‐GlcNAcylation and NF‐κB p65 O‐GlcNAcylation and its nuclear retention in LRP1‐silenced HCC cells. Despite no influence on OGA mRNA levels (Figure 4a), LRP1 deletion markedly accelerated ubiquitin‐dependent OGA degradation, whereas β∆‐chain overexpression significantly slowed down this process. These results add to the growing body of evidence showing the importance of the tumor‐promoting role of O‐GlcNAcylation, and reveal a novel link between LRP1 and NF‐κB O‐GlcNAcylation converging on OGA.

Given that DAPT administration and β∆‐chain overexpression counterregulated cellular O‐GlcNAcylation levels, we hypothesized that LRP1‐ICD derived from LRP1 cleavage may increase the expression of a specific deubiquitinase or decrease the expression of a specific ubiquitinase that affects the stability and degradation of OGA, thereby affecting global O‐GlcNAcylation levels and HCC formation. Although mounting evidence suggests that OGT stability can be regulated by the ubiquitin‐proteasome system, the literature investigating OGA degradation is sparse.^[^ ^26^ ^]^ Here, we showed that LRP1 deficiency triggered UFL1 to interact with OGA, subsequently promoting the ubiquitylation and degradation of OGA, whereas the exogenously expressed β∆‐chain maintained the protein stability of OGA, thereby restoring normal O‐GlcNAcylation levels by disrupting the association between UFL1 and OGA. Despite the lack of characteristic HETC and RING domains, UFL1 functions as the only known E3 ligase in the ubiquitin‐like modifier 1 (UFM1) modification system that conjugates UFM1 to target proteins.^[^ ^27^ ^]^ Our results are consistent with a recent study showing that UFM1 modification (UFMylation) of programmed cell death protein 1 (PD‐L1) reduced PD‐L1 stability by synergizing its ubiquitination, whereas knockout of UFL1 prevented proteasome‐mediated degradation of PD‐L1, implying potential crosstalk between UFMylation and ubiquitination.^[^ ^14^ ^]^ By contrast, UFMylation of both estrogen receptor‐α and P53 could inhibit their ubiquitination.^[^ ^28^ ^]^ Therefore, it would be interesting to explore how UFMylation, together with de‐UFMylation, tightly regulates protein ubiquitination to modulate various physiological and pathophysiological processes. Although UFL1 expression was unaltered in the absence of LRP1, our results demonstrated that silencing of UFL1 exerted anti‐tumor effects in HCC cells with LRP1 knockdown by consequent reactions: 1) reducing cellular O‐GlcNAcylation levels through stabilization of OGA; 2) attenuating NF‐κB p65 O‐GlcNAcylation; 3) promoting apoptosis of HCC cells by inhibiting Bcl‐2 and enhancing Bax expression; and 4) suppressing the proliferation and migration abilities of HCC cells. Notably, UFL1 has been reported to function as a negative regulator of NF‐κB signaling, inhibit NF‐κB activity, and prevent LPS‐induced ER stress and apoptosis.^[^ ^29^ ^]^ Although aberrant UFM1 modification has been implicated in various types of human cancers, its biological role of UFL1 in tumorigeneses remains to be investigated.^[^ ^30^ ^]^ In view of the diverse functions of UFL1, further studies are needed to elucidate the detailed mechanism by which UFL1 coordinates with LRP1 to regulate OGA turnover and the downstream activation of NF‐κB.

## 4 Conclusion

**Figure 7:**
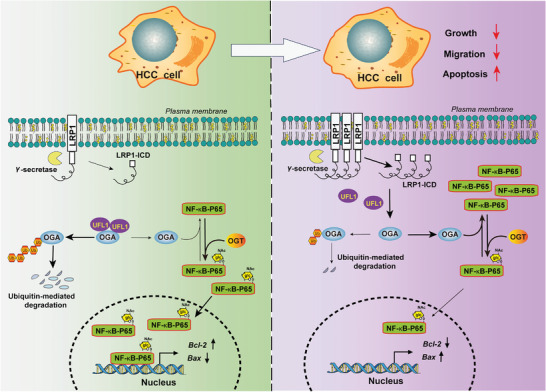
A model describing that the loss of LRP1 expression promotes HCC tumorigenesis by facilitating UFL1 binding to OGA and increasing ubiquitination and degradation of OGA, whereas LRP1‐ICD derived from the cleavage of the exogenously expressed β‐chain restores the protein stability of OGA, thereby attenuating HCC progression by disrupting the association between UFL1 and OGA.

In summary, our data indicate that reduced LRP1 expression promotes malignant transformation of HCC, and that accelerated OGA degradation and elevated NF‐κB p65 O‐GlcNAcylation is causal to this. Moreover, we uncover a novel regulatory role of LRP1, especially its β‐chain, in modulating the association between UFL1 and OGA to maintain OGA stability and exert tumor‐suppressing activity by abating NF‐κB O‐GlcNAcylation and inhibiting its downstream signaling pathway (**Figure** **7**). These findings substantially advance our understanding of the relevance of OGA and NF‐κB post‐translational modifications, and the important role of LRP1 in the pathogenesis of HCC. Given the striking anti‐tumor effects on HCC cells and hepatoma xenografts, the LRP1 β∆‐chain, an endogenous short peptide, may be a promising anti‐cancer agent with an optimal safety profile and high tolerability for use in patients with HCC.

## 5 Experimental Section

### Database Source and Data Analysis

The LRP1 expression profiles and related clinical pathological data were downloaded from the Gene Expression Omnibus (GEO) datasets (http://www.ncbi.nlm.nih.gov/geo) and TCGA‐LIHC dataset (https://ualcan.path.uab.edu), respectively. The histological grade and survival probability were analyzed in HCC patients with different LRP1 expression levels. The threshold for difference analysis was set at *p*‐value < 0.05.

### Clinical Samples

HCC tissue samples and paired adjacent normal liver tissues were obtained from patients who underwent resection operation at the Second Affiliated Hospital of Chongqing Medical University between Jan. 2019 and Dec. 2021. HCC patients with hepatitis B virus infection or any other malignant tumor were excluded from this study, and the clinical information for all patients (*n* = 30) involved in the study is shown in Table S1 (Supporting Information).

### Antibodies and Reagents

The antibodies and reagents used are listed in Table S2 (Supporting Information).

### Cell Culture

HEK293T and human HCC cell lines were maintained in DMEM culture medium supplemented with 10% fetal bovine serum (FBS) and 1% antibiotics at 37 °C with 5% CO_2_.

### Plasmids Construction and Molecular Cloning

The gene fragment encoding the α‐chain (amino acids 20–3943), β‐chain (3944‐4544), and truncated β∆‐chain (4195‐4544) of human LRP1 was amplified from the *hLrp1*‐pcDNA3.1 plasmid by PCR and cloned into the plasmid pcDNA 3.1‐3×Flag using the In‐Fusion Ready‐to‐Use Seamless cloning kit to express the Flag‐tagged β‐chain. The pCS2‐Flag‐OGA plasmid was a gift from Dr. Haishan Gao (Westlake University, Hangzhou, China). The pcDNA3.1‐UFL1‐HA plasmid was purchased from Youbao Biotechnology Co., Ltd. (Changsha, China). All constructs were verified by DNA sequencing and western blotting. The oligonucleotide sequences used for cloning are listed in Table S3 (Supporting Information).

### Lentivirus Production and Infection

The pLKO.1 lentiviral vector carrying short hairpin RNA (shRNA) was generated as previously described.^[^ ^31^ ^]^ The shRNA sequence targeting the 3′‐untranslated region (3′‐UTR) of the human *Lrp1* gene was as follows: CCGGCGCCGGATGTATAAATGTAAACTCGAGTTTACATTTATACATCCGGCGTTTTTG. The scrambled shRNA (CCTAAGGTTAAGTCGCCCTCGCTCGAGCGAGGGCGACTTAACCTTAGG) was a gift from David Sabatini.^[^ ^32^ ^]^ For lentivirus‐mediated overexpression, the β∆‐chain‐coding fragment was ligated into the lentiviral vector pHAGE‐CMV‐MCS‐IZsGreen. The lentiviral vector particles were generated following the manufacturer's instructions. HEK293T cells were co‐transfected with a lentiviral packaging mix (psPAX2, pMD2.G) and lentiviral plasmid using FuGENE 6 Transfection Reagent. Viral particle‐containing supernatants were harvested 48–72 h after transfection, centrifuged, and stored at −80 °C until use.

### sWGA Pull‐Down Assay

Tumor tissues or MHCC‐97H cells were lysed in lysis buffer (125 mM NaCl, 50 mM Tris pH 7.4, 5 mM EDTA, and 0.1% NP‐40) supplemented with protease and phosphatase inhibitors. The cell lysate supernatant was denatured in glycoprotein‐denaturing buffer and digested with PNGase to remove N‐linked glycoproteins, followed by overnight incubation with sWGA‐conjugated agarose beads at 4 °C. The monosaccharide inhibitor N‐Acetyl‐D‐glucosamine (GlcNAc, 20 mM) was used as the negative control. The precipitated complexes were washed, eluted, and subjected to immunoblotting analysis using the indicated antibodies.

### Cell Proliferation Assay

Cells were cultured overnight in 96‐well microplates at a density of 2.0 × 10^3^/well and cell viability assay was performed using CCK8 (Beyotime, Shanghai, China) following the manufacturer's instructions. For colony formation, cells were seeded in 6‐well plates at a density of 300 cells/well. The medium was changed in 48 h after transfection and then twice a week for 10–15 days. Colonies were fixed and stained with Crystal Violet, and images were captured using a Nikon NI‐U digital camera. The percentage of coverage area was calculated using ImageJ software.

### Wound Healing Assay

Cells were seeded in 12‐well culture plates at a density of 1 × 10^5^/well, and scratched when they reached≈90% confluency. After washing with PBS, cells were incubated in FBS‐free medium for 48 h. Images were captured at 0 and 48 h, and the migration rate was analyzed using the ImageJ software.

### Cell Apoptosis Assay

After 48 h of culture, the cells were trypsinized, washed, and stained using an Annexin V/PI detection kit (4A Biotech, Beijing, China) for 5 min at room temperature. Apoptotic cells were counted by flow cytometry (Beckman Coulter).

### Subcutaneous HCC Tumor Model

Cultured MHCC‐97H cells were resuspended in serum‐free DMEM at a cell density of 5 × 10^6^/mL and injected subcutaneously into the right flank regions of male BALB/C nude mice aged 4 weeks. Tumor size was measured with calipers every two days. After 4 weeks of xenograft implantation, the nude mice were sacrificed and the tumor masses were surgically removed and weighed.

### Orthotopic HCC Tumor Model

Approximately, 1 × 10^6^ MHCC‐97H cells were resuspended in a mixture of serum‐free DMEM and Matrigel (1:2) and orthotopically implanted into the livers of 5‐week‐old male BALB/C nude mice after anesthesia. Mice were sacrificed for further analysis 30 days after intrahepatic injection.

### Immunoblot and Co‐Immunoprecipitation (Co‐IP)

Cell lysates and tissue homogenates were separated by SDS‐PAGE and immunoblotted with the indicated antibodies. Co‐IP for exogenously expressed proteins, the transfected cells were lysed and subjected to FLAG immunoprecipitation using anti‐Flag M2 affinity gel. The immune complexes were eluted with 3× Flag peptide proteins and subjected to mass spectrometry and western blotting analysis. For endogenous OGA immunoprecipitation experiments, the cell lysates were incubated with anti‐OGA antibodies followed by the addition of protein A/G‐Sepharose beads. The beads were washed, eluted, and subjected to SDS‐PAGE and immunoblotting with the indicated antibodies (Table S2, Supporting Information).

### HE and Immunohistochemistry (IHC)

Formalin‐fixed, paraffin‐embedded tissues were stained with hematoxylin and eosin (H&E) and immunohistochemically stained as described previously using the indicated antibodies.^[^ ^33^ ^]^ Five representative regions from each section were photographed at 200× magnification using a Nikon NI‐U digital camera (Japan).

### Statistical Analysis

The results were reported as mean ± SEM. The unpaired 2‐tailed Student's t‐test was used to compare differences between the two groups with GraphPad Prism (version 5.0). Differences among means of three or more groups were analyzed using one‐ or two‐way ANOVA with Tukey's or Sidak's post‐hoc multiple comparison tests. The Pearson's correlation coefficient was used to test for linear correlations. Differences were considered statistically significant at *p* < 0.05.

### Ethics Approval and Consent to Participate

All patients involved in the study offered written informed consent according to protocols approved by the Ethics Committee of Chongqing Medical University (Approval No.2023015). All animal studies were conducted at the Animal Institute of Chongqing Medical University according to protocols approved by the Institutional Animal Use and Care Committee of the University (Approval No. IACUC‐CQMU‐2023‐0058).

### Consent for Publication

All authors have provided their consent for the publication of this manuscript.

## Conflict of Interest

The authors declare that they have no competing interests.

## Author Contributions

X. G., F.Y., T.L., A.C., and D.L. contributed equally to this work. All authors contributed to conceptualization (X.G., F.Y., T.L., A.C., and Y.D.), data acquisition (X.G., F.Y., T.L., A.C., D.L., J.P., C.J., Y.W., and J.Y.), data analysis and interpretation (X.G., F.Y., T.L., A.C., D.L., J.P. and C.J.), statistical analysis (Y.W., J.Y., and N.O.), and original draft (X.G., F.Y., T.L., A.C., and D.L.). J.H. reviewed the assembled data and advised on the revision of the manuscript. Y.D. supervised the study and revised the manuscript. All the authors have read and approved the final version of the manuscript.

## Supporting information



Supporting Information

## Data Availability

The data generated in this study are publicly available in the GEO repository (http://www.ncbi.nlm.nih.gov/geo/), TCGA‐LIHC dataset (https://ualcan.path.uab.edu), and within the article and its supplementary data files. The raw data supporting the findings of this study are available from the corresponding author upon request.
