# Supplementary material for: Loss of LRP1 Promotes Hepatocellular Carcinoma Progression via UFL1‐Mediated Activation of NF‐κB Signaling
Source: Adv Sci (Weinh). 2024 Oct 15;11(45):2401672. doi: 10.1002/advs.202401672 (PMC11615765; doi:10.1002/advs.202401672)
Supplement: Supplementary file 1 — Supporting Information [file ADVS-11-2401672-s001.docx]

**Supporting Information**

Loss of LRP1 Promotes Hepatocellular Carcinoma Progression via UFL1-Mediated Activation of NF-κB Signaling

*Xingxian Guo,* *Fan Yang,* *Tianyi Liu, Amei Chen,* *Dina Liu,* *Jiangxia Pu,* *Can Jia,* *Yuanhong Wu,* *Junfeng Yuan,* *Nan Ouyang, Joachim Herz,* *and* *Yinyuan Ding**

**
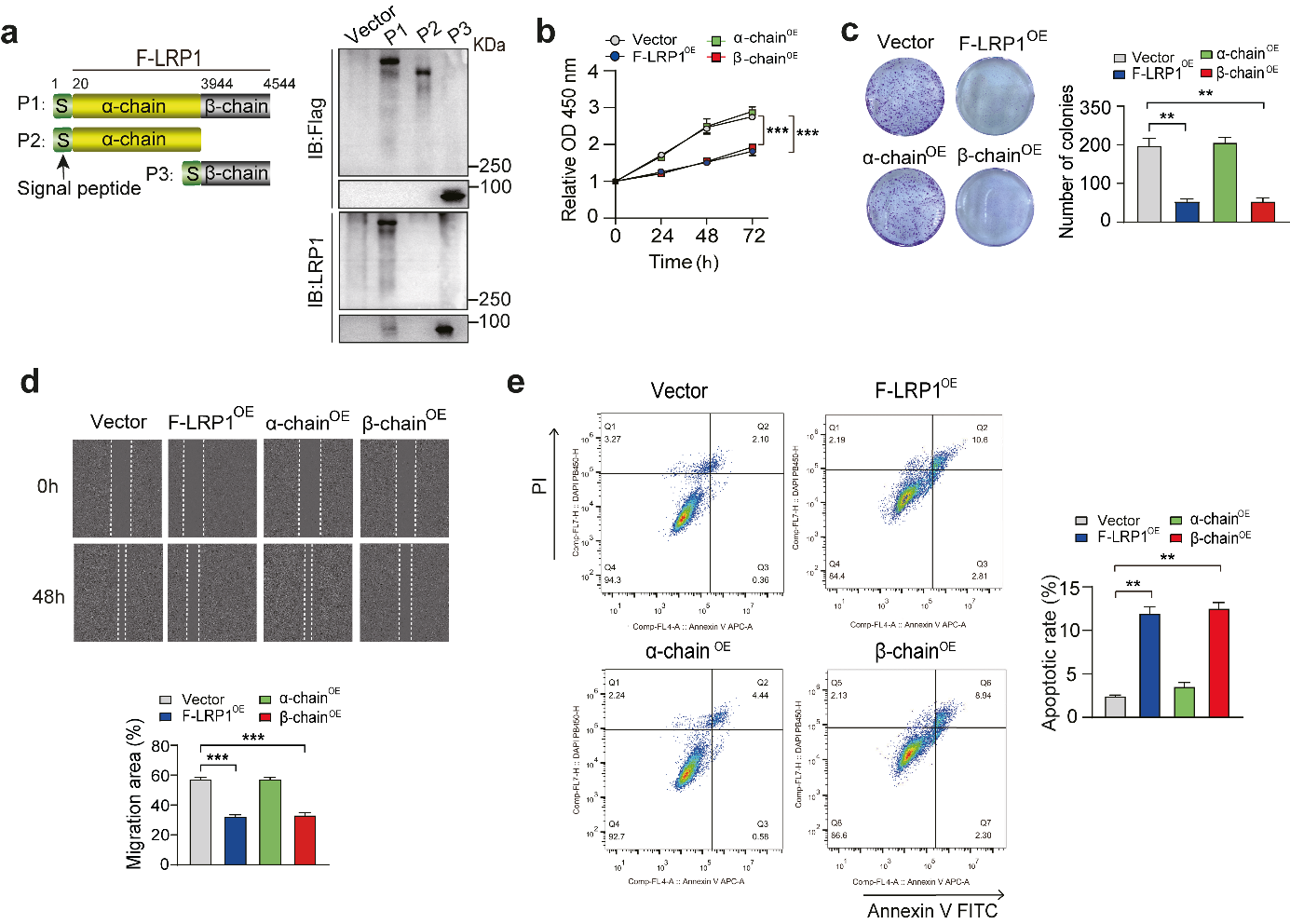
**

**Figure S1.** The effect of LRP1 on the proliferation and migration of Huh-7 cells. a) Schematic illustrating the generation of constructs carrying full-length LRP1 (F-LRP1) or its α- or β-chain coding sequences and western blot analysis of Flag-tagged protein and LRP1 expression in Huh-7 cells expressing F-LRP1 (P1), α- (P2), and β-chain (P3), respectively. b-c) CCK-8 and colony formation assay in Huh-7 cells transfected with the indicated plasmids. d) Wound healing assay in Huh-7 cells at 48h after indicated treatment, e) FACS analysis in the Huh-7 cells under indicated treatment. Data are mean ± SEM. n = 5 for each group (b-c); n = 3 for each group (d-e). Statistical analyses were performed using two-way ANOVA (b) or one-way ANOVA (c-e) with Tukey’s multiple comparisons test. ** *p* < 0.01, *** *p* < 0.001.


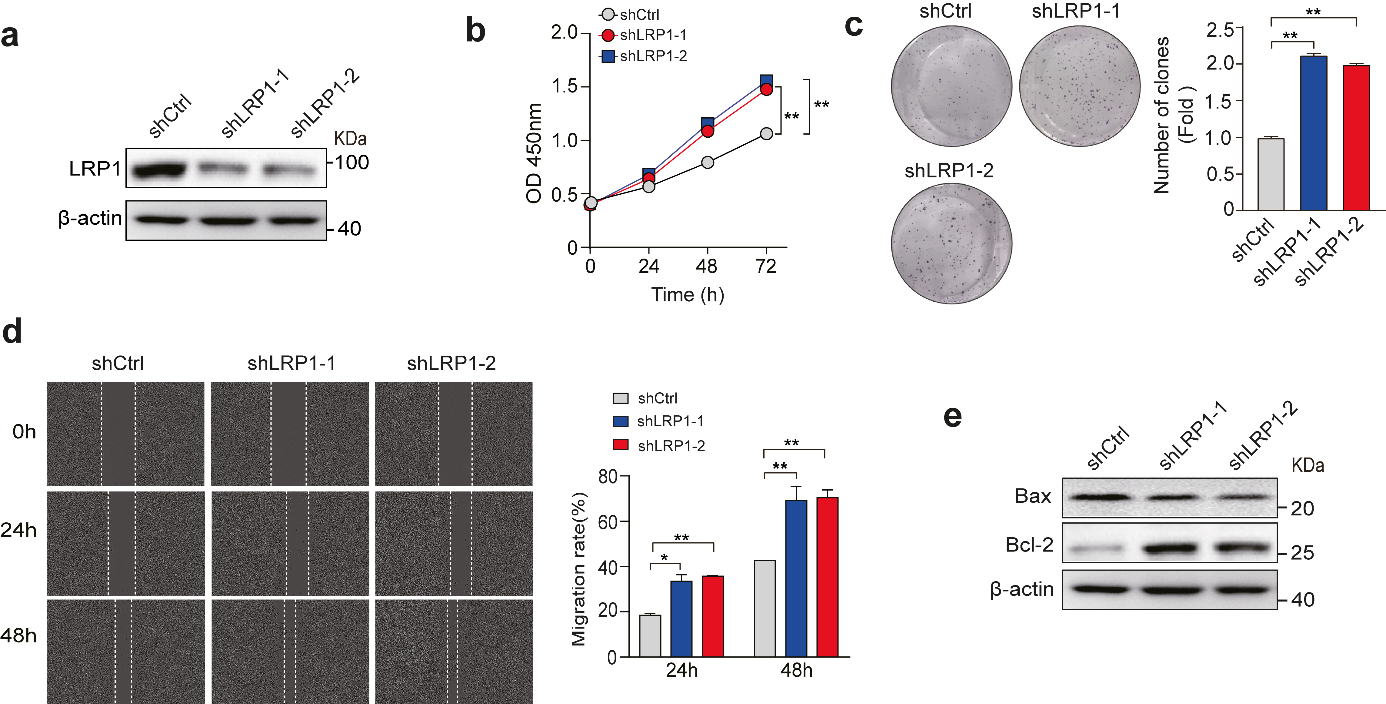


**Figure S2.** LRP1 silencing promotes tumorigenesis in HepG2 cells. a) Representative western blots for LRP1 expression in HepG2 cells treated with shCtrl, LRP1 shRNA-1 (shLRP1-1), or LRP1 shRNA-2 (shLRP1-2) lentivirus. b-c) Evaluation of proliferation capacity using CCK-8 (b) and colony formation (c) assays. d) Wound healing assay. e) Western blot analysis of apoptosis-related protein expression. Data are mean ± SEM. n = 5 independent experiments for panels b-c; n = 3 independent experiments for panel d. Statistical analyses were performed using two-way ANOVA (b) or one-way ANOVA (c-d) with Tukey’s multiple comparisons test. * *p* < 0.05, ** *p* < 0.01.


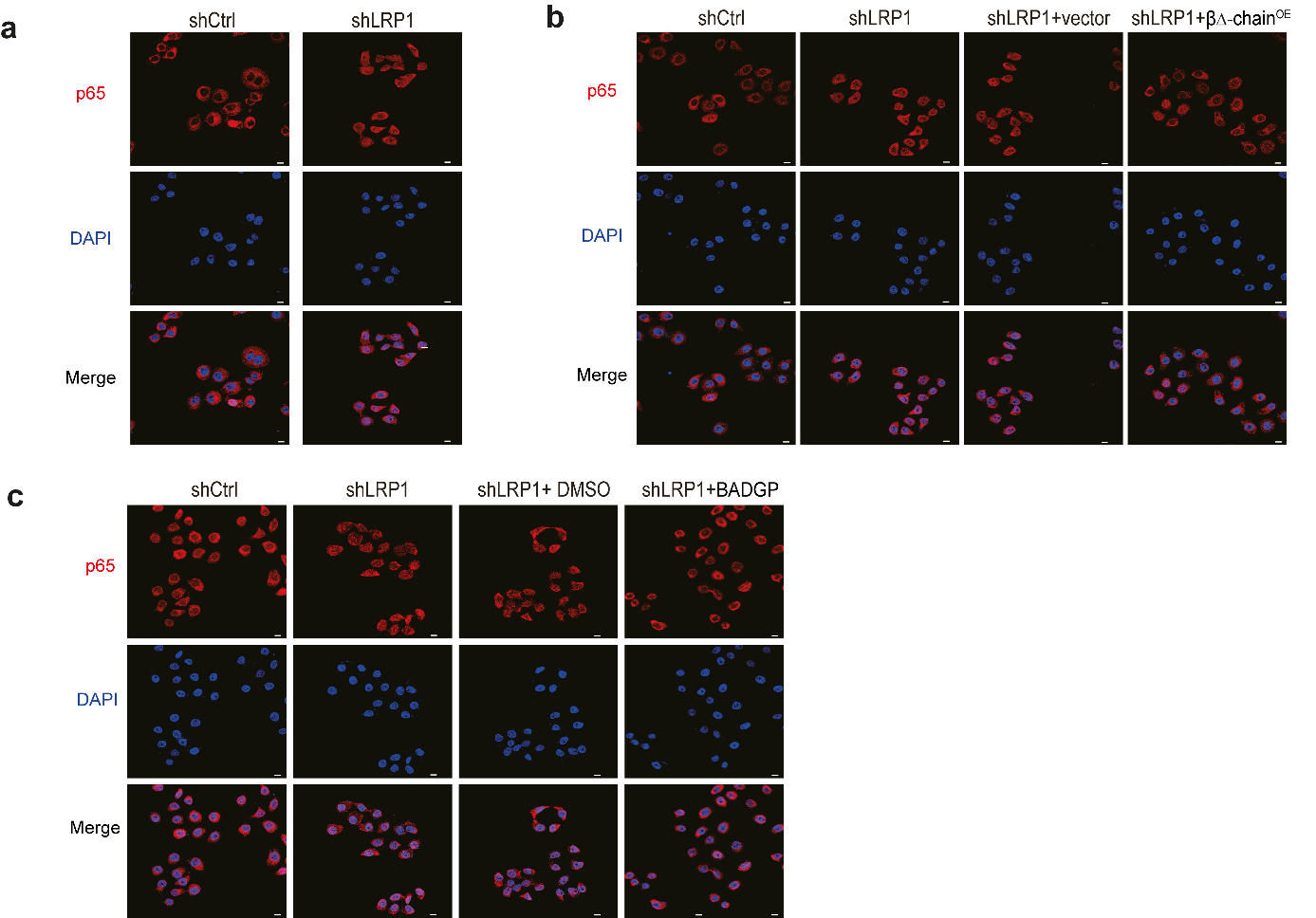


**Figure S3.**  LRP1 affects the subcellular distribution of NF-κB. a) MHCC-97H cells with and without LRP1 knockdown were immunofluorescenced with NF-κB p65 antibody and analyzed by confocal microscopy. b) The subcellular localization of NF-κB p65 in shCtrl and shLRP1 MHCC-97H cells overexpressing with or without the β∆-chain. c) Assessment of NF-κB p65 localization in shCtrl and shLRP1 MHCC-97H cells treated with or without 5 mM BADGP for 24h. Images are representative of three independent experiments. Scale bar, 10 µm.

**
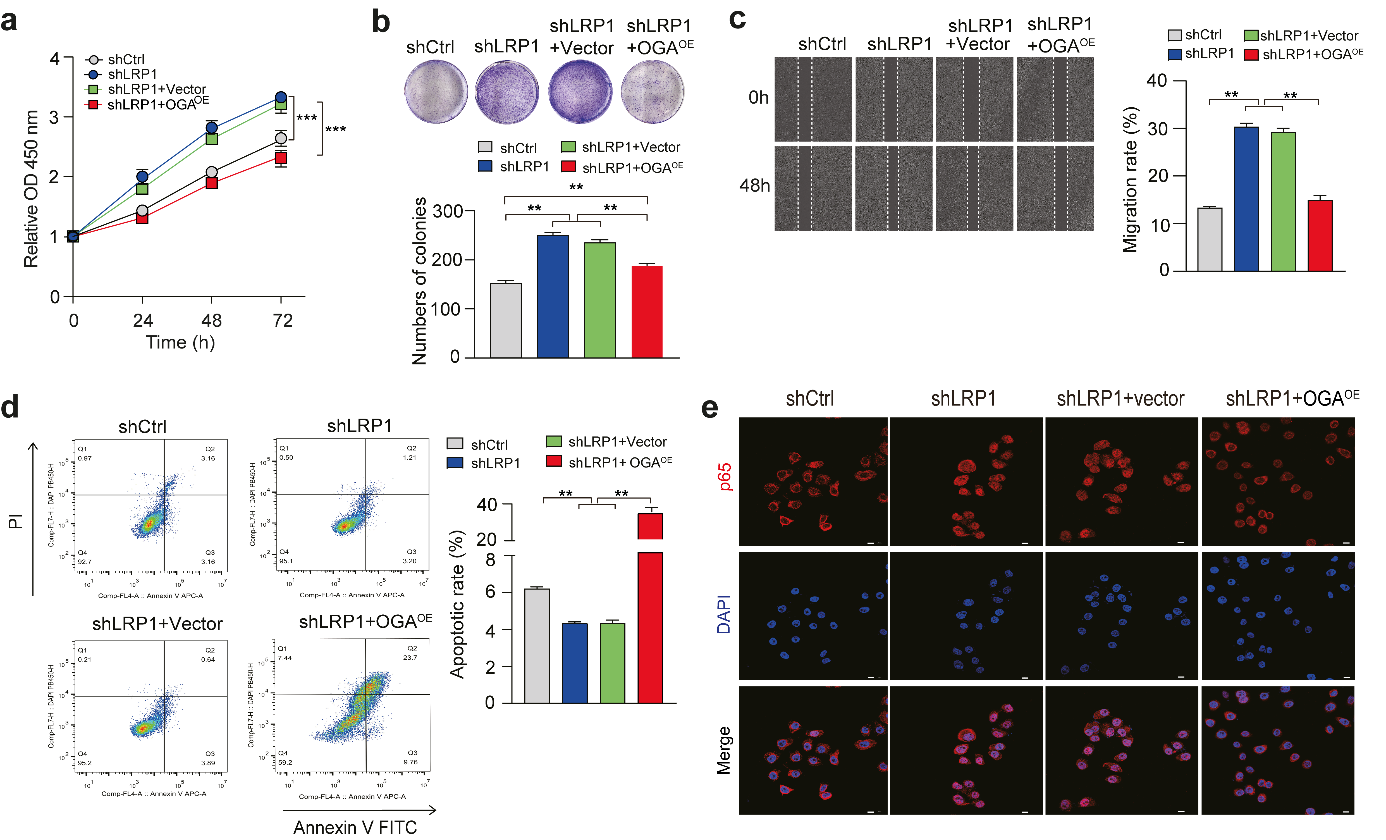
**

**Figure S4.** OGA overexpression blocks the effects of LRP1 deficiency on tumorigenicity of HCC cells. a-b) CCK-8 (a) and colony formation (b) assays in shCtrl and shLRP1 MHCC-97H cells with or without OGA overexpression. c) Wound healing assay. d) FACS analysis. e) Representative confocal fluorescence images of the NF-κB p65 (red) in MHCC-97H cells. The nuclei were counter-stained with DAPI (blue). Scale bar, 10 µm. Images are representative of three independent experiments. Data are mean ± SEM. n = 5 independent experiments (a-b); n = 3 independent experiments (c-d). Statistical analyses were performed using two-way ANOVA (a) or one-way ANOVA (b-d) with Tukey’s multiple comparisons test. ** *p* < 0.01, *** *p* < 0.001.

**
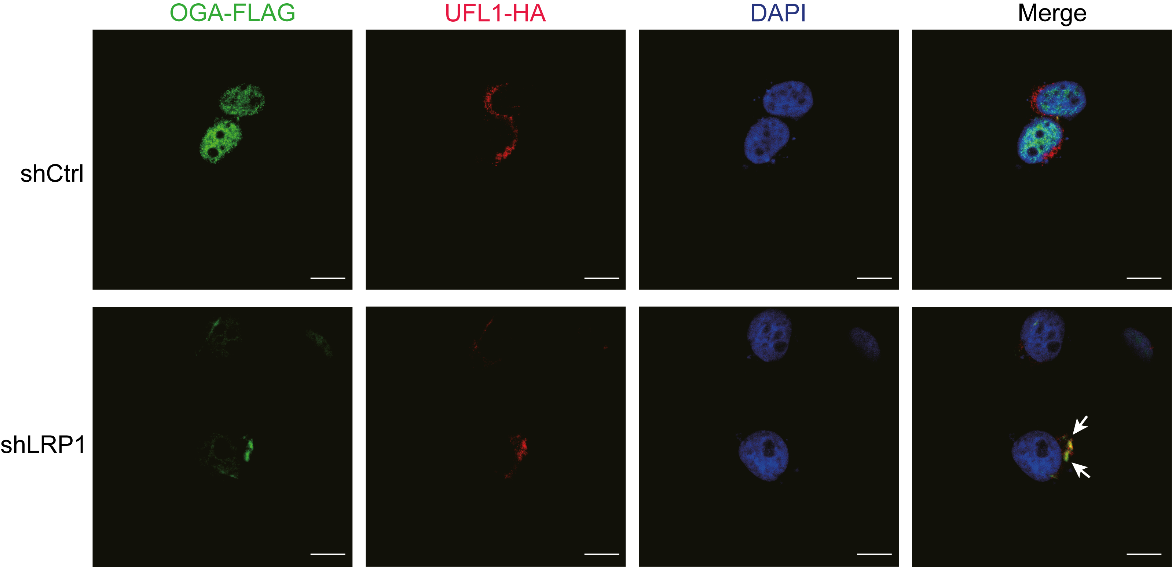
**

**Figure S5.**  LRP1 knockdown promotes colocalization of UFL1 and OGA. a) The shCtrl and shLRP1 knockdown MHCC-97H cells overexpressing Flag-tagged OGA and HA-tagged UFL1 were immunostained with anti-Flag and anti-HA antibodies and analyzed by confocal microscopy. Scale bar, 5 μm. Images are representative of three independent experiments.


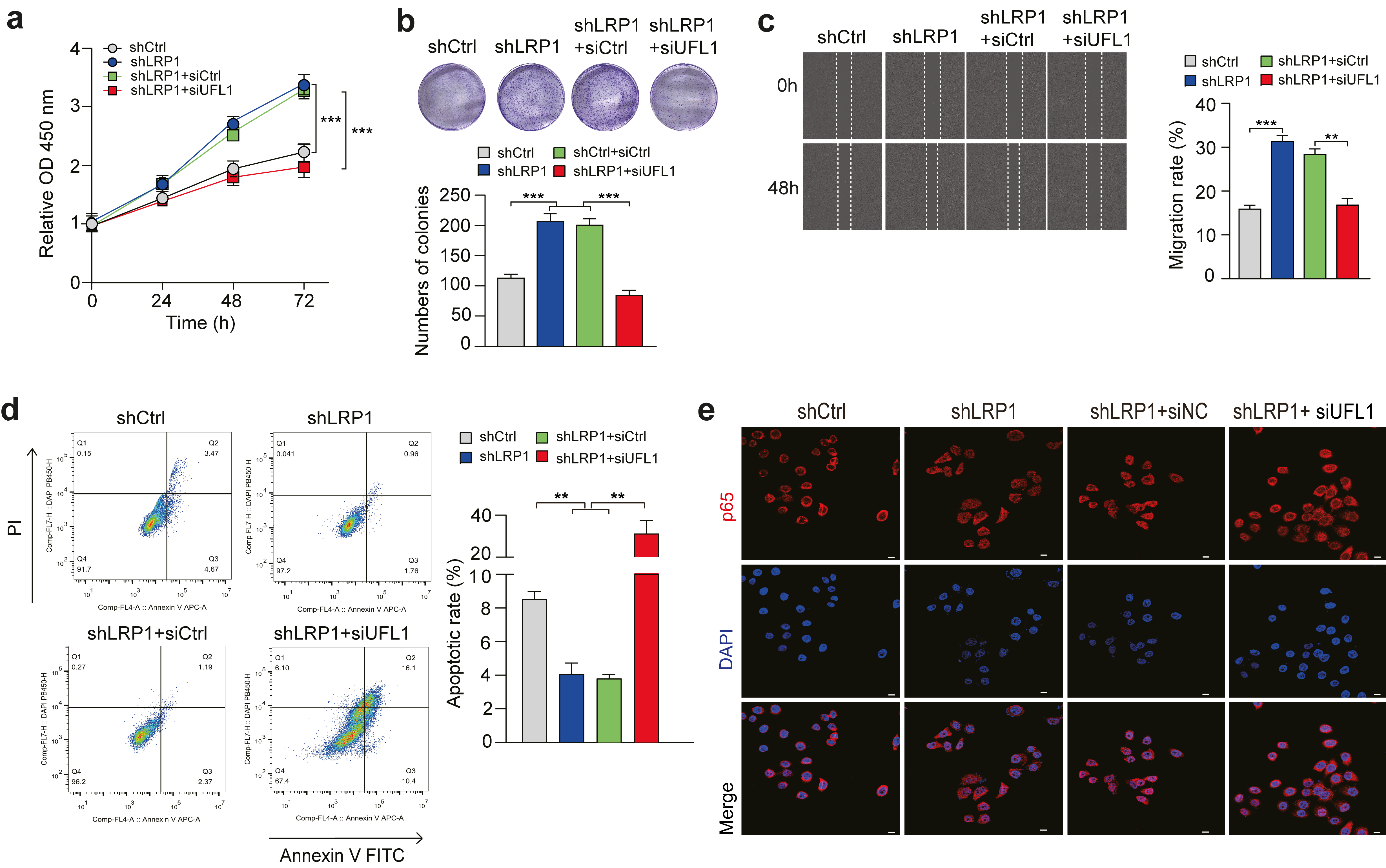


**Figure S6.** Silencing of UFL1 blocks the tumor-promoting effects of LRP1 knockdown in MHCC-97H cells. a-b) CCK-8 kit (a) and colony formation (b) assays in shCtrl- and shLRP1-lentivirus infected MHCC-97H cells with or without UFL1 silencing. c) Wound healing assay. d) FACS analysis. e) Representative confocal fluorescence images of the NF-κB p65 (red) in shCtrl and shLRP1 knockdown MHCC-97H cells transfected with siCtrl or siUFL1. The nuclei were counter-stained with DAPI (blue). Scale bar, 10 µm. Images are representative of three independent experiments. Data are mean ± SEM. n = 5 independent experiments (a-b); n = 3 independent experiments (c-d). Statistical analyses were performed using two-way ANOVA (a) or one-way ANOVA (b-d) with Tukey’s multiple comparisons test. ** *p* < 0.01, *** *p* < 0.001.


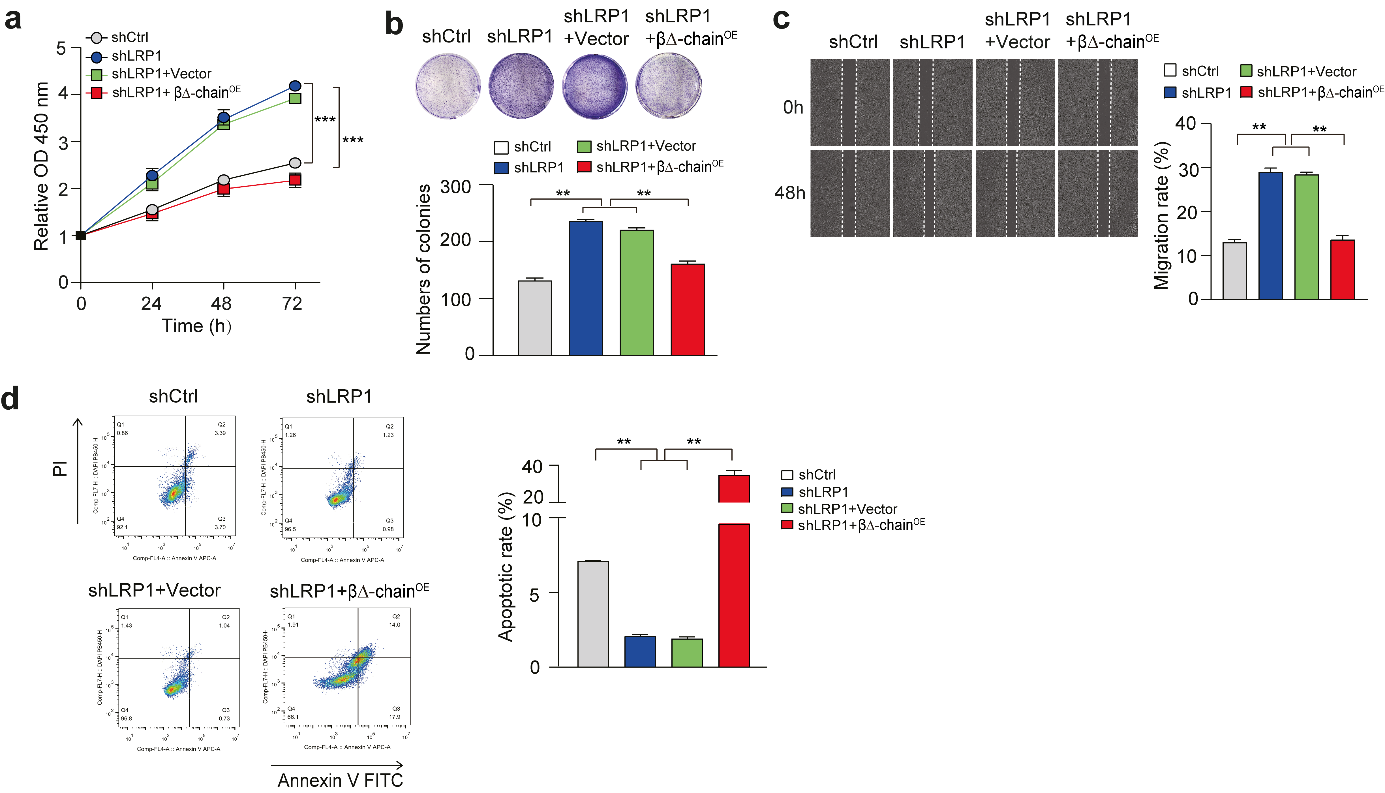


**Figure S7.** LRP1 β∆-chain overexpression inhibits the tumor-promoting effects of LRP1 deficiency in MHCC-97H cells. a-b) CCK-8 (a) and colony formation (b) assays in shCtrl and shLRP1 cells with or without LRP1 β∆-chain overexpression. c) Wound healing assay in MHCC-97H cells under the indicated treatment. d) FACS analysis. Data are mean ± SEM. n = 5 independent experiments (a-b); n = 3 independent experiments (c-d). Statistical analyses were performed using two-way ANOVA (a) or one-way ANOVA (b-d) with Tukey’s multiple comparisons test. ** *p* < 0.01, *** *p* < 0.001.

**
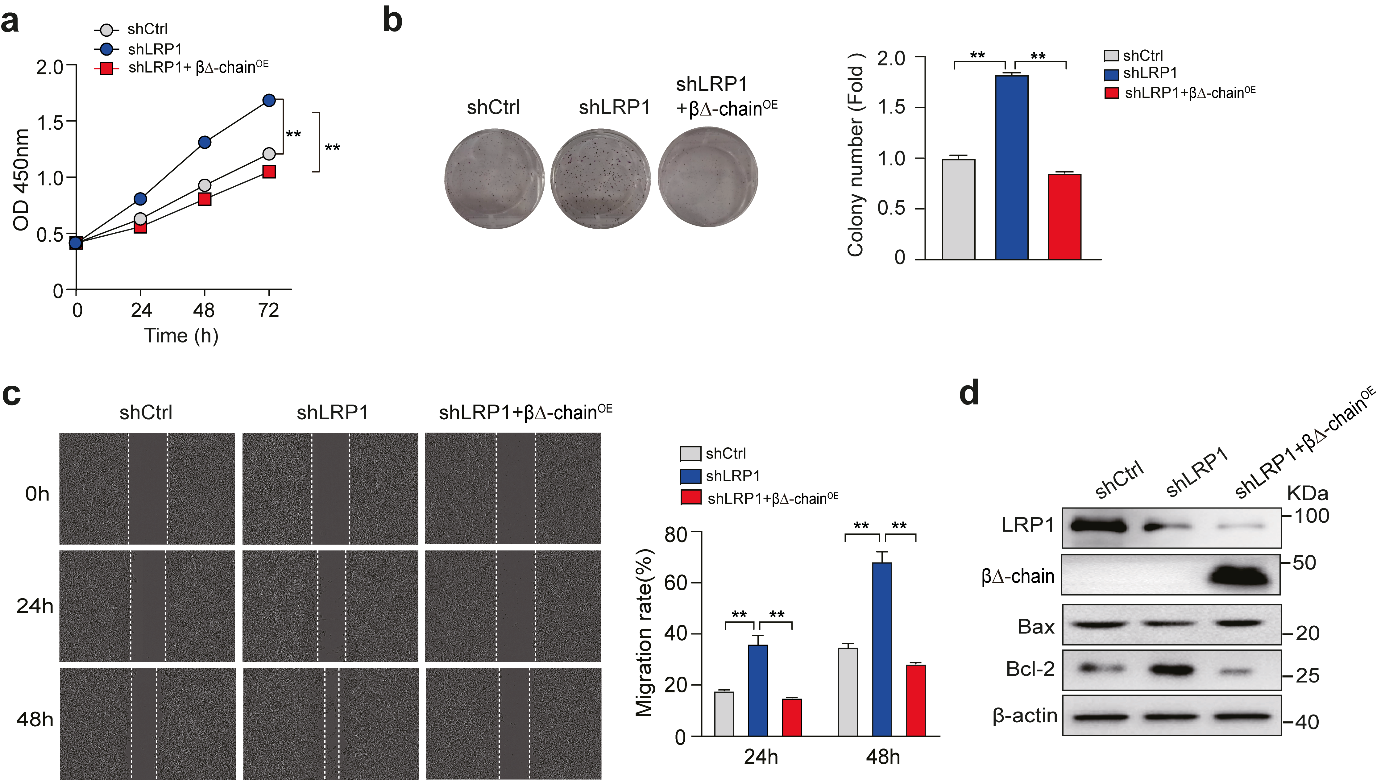
**

**Figure S8.** Overexpression of the LRP1 β∆-chain reverses the pro-tumor effects of LRP1 knockdown in HepG2 cells. a-b) CCK-8 kit (a) and colony formation (b) assays in LRP1-deleted HepG2 cells transfected with control or β-chain plasmid. c) Wound healing assay in shCtrl and shLRP1 HepG2 cells with or without β∆-chain overexpression. d) Western blot analysis of apoptosis-related protein expression in HepG2 cells under the indicated treatment. Data are mean ± SEM. n = 5 independent experiments (a); n = 3 independent experiments (b-c). Statistical analyses were performed using two-way ANOVA (a) or one-way ANOVA (b, c) with Tukey’s multiple comparisons test. ** *p* < 0.01.

**
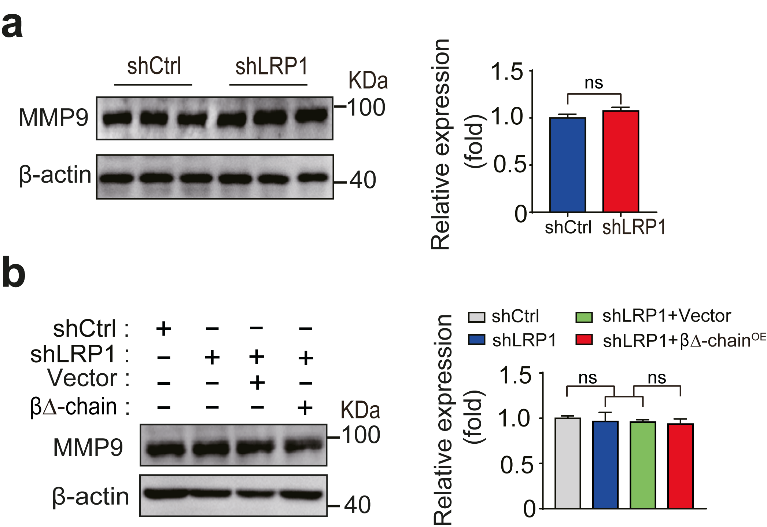
**

**Figure S9.** The expression of MMP9 in MHCC-97H cells. a) Expression of MMP9 in MHCC-97H cells with and without LRP1 knockdown. b) The expression of MMP9 in shCtrl and shLRP1 MHCC-97H cells overexpressing with or without the β∆-chain. Data are mean ± SEM. n = 3 independent experiments (b). Statistical analyses were performed using unpaired 2-tailed Student's t-test (a), or one-way ANOVA with Tukey’s multiple comparisons test (b). ns indicates no significant differences.

**Table S1.** Clinicopathological Features of the Patients with HCC.

| **Case** | **Sex** | **Age** | **Tumor size (cm)** | **AFP** | **HBsAg** | **Grade** | **Cirrhosis** | **MVI** | **Chil-pugh score** |
| --- | --- | --- | --- | --- | --- | --- | --- | --- | --- |
| 1 | F | 50 | 9 | 26.5 | + | II | - | - | A |
| 2 | M | 45 | 3.5 | 48.6 | + | II | + | + | B |
| 3 | M | 47 | 10 | 316.3 | + | III | + | + | C |
| 4 | M | 57 | 7.5 | 3.4 | + | II | - | - | A |
| 5 | M | 70 | 9 | 3064 | + | I | - | - | A |
| 6 | M | 48 | 4 | 8875 | + | II | + | + | B |
| 7 | F | 76 | 4.6 | 45 | - | I | - | - | A |
| 8 | M | 72 | 9 | 2.4 | - | II | + | - | B |
| 9 | M | 55 | 5.5 | 2437 | + | I | - | + | A |
| 10 | M | 59 | 11 | 17.9 | + | I | + | - | A |
| 11 | M | 46 | 7.5 | 18.5 | + | III | + | + | B |
| 12 | M | 65 | 10 | 2.8 | + | II | + | + | A |
| 13 | F | 71 | 7 | 8.7 | - | II | + | - | A |
| 14 | M | 51 | 5.3 | 84596 | + | I | - | + | A |
| 15 | M | 65 | 9.5 | 38887 | + | I | - | - | A |
| 16 | F | 64 | 6 | 2472 | + | II | + | + | B |
| 17 | M | 52 | 5 | 87.7 | + | I | - | - | A |
| 18 | M | 68 | 8.5 | 12.8 | - | III | + | + | B |
| 19 | M | 51 | 14 | 1539 | + | II | - | - | A |
| 20 | M | 66 | 8 | 3.7 | + | I | + | - | B |
| 21 | M | 64 | 13 | 82849 | + | I | + | - | A |
| 22 | M | 60 | 5 | 24 | + | I | + | - | A |
| 23 | M | 67 | 9 | 48.6 | + | III | + | + | B |
| 24 | M | 65 | 10.9 | 503 | + | I | - | - | A |
| 25 | M | 53 | 10 | 205 | + | I | - | + | A |
| 26 | M | 60 | 11 | 64.6 | - | II | - | + | A |
| 27 | M | 49 | 9 | 616.9 | + | II | + | + | B |
| 28 | M | 62 | 6 | 108.1 | - | I | - | + | A |
| 29 | M | 74 | 10 | 338 | - | III | + | + | B |
| 30 | M | 53 | 12 | 5.1 | + | I | - | - | A |

Abbreviation: MVI, microvascular invasion

**Table S2.** Reagent or resource.

**REAGENT or RESOURCE SOURCE IDENTIFIER**

**Antibodies**

| Rabbit anti-OGT | Abcam | Cat # ab177941 | |
| --- | --- | --- | --- |
| Rabbit anti-OGA/MGEA5 | Abcam | Cat # ab124807 | |
| Rabbit anti-GFPT1 | Abcam | Cat # ab125069 | |
| Mouse anti-O-GlcNAc | Abcam | Cat # ab2739 | |
| Rabbit anti-MMP9 | Abcam | Cat # ab38898 | |
| Rabbit anti-β-actin | Proteintech | Cat # 81115-1-RR | |
| Mouse anti-Ubiquitin | Santa Cruz | Cat # sc-166553 | |
| Rabbit anti-Bax | Cell Signaling Technology | Cat # 41162S | |
| Rabbit anti-Bcl-2 | Cell Signaling Technology | Cat # 3498 | |
| Goat anti-rabbit IgG (H+L) | Abcam | Cat # ab6721 | |
| Goat anti-mouse IgG | Abcam | Cat # ab6789 | |
| Rabbit anti-LRP1 | Selleck | Cat # A5229 | |
| Rabbit anti-NF-κB p65 | Cell Signaling Technology | Cat # 8242 | |
| Rabbit anti-UFL1 | Proteintech | Cat # 26087-1-AP |  |
| Rabbit-anti-Calnexin | Beyotime | Cat # AF2425 |  |
| Rabbit-anti-Flag | Merck | Cat # F7425 |  |
| HA-tag Polyclonal antibody | Proteintech | Cat # 51064-2-AP |  |
| Rabbit-anti-Histone-H3 | Cell Signaling technology | Cat # 9715 |  |

**Bacterial and Virus Strains**

DH5a Competent E.coli Lab stock

LRP1 shRNA lentivirus-1 This paper

LRP1 shRNA lentivirus-2 This paper

LRP1 β-chain lentivirus This paper

**Biological Samples**

Human liver tissue The Second Affiliated Hospital

Chongqing Medical University. China

**Chemicals, peptides, and Recombinant Proteins**

| Fetal Bovine Serum | Excell | Cat # FSS500 |
| --- | --- | --- |
| Trypsin | Gibco | Cat #12605010 |
| Opti-MEM^TM^ Reduced Serum Medium | Gibco | Cat # 31985070 |
| DMEM | Gibco | Cat #11995500BT |
| Penicillin-Streptomycin Solution | HyClone | Cat # SV30010 |
| DMSO | Sigma | Cat # D2660 |
| Cycloheximide | MCE | Cat # HY-12320 |
| DAPT | MCE | Cat # HY-13027 |
| BADGP | Sigma | Cat # B4894 |
| Pierce™ protein A/G magnetic beads  Anti-FLAG M2 Affinity Gel  3xFlag peptide  FuGENE 6 Transfection Reagent | Thermo  Merck  Merck  Promega | Cat # 88803  Cat # A2220  Cat # F4799  Cat # E2691 |
| N-Acetyl-D-glucosamine | Sigma-Aldrich | Cat # A8625 |
| sWGA | Vectorlabs | Cat # AL-1023S-2 |
| PNGase F | NEB | Cat # P0709S |
| PrimeSTAR® Max DNA Polymerase | Takara | Cat # R045Q |
| Ready-to-Use Seamless Cloning Kit | Sangon | Cat # B632219 |

Matrigel Matrix Basement Membrane Corning Cat # 354234

**Experimental Models: Cell Lines**

HEK293T Lab stock

MHCC-97H Lab stock

HepG2 Lab stock

Huh-7 Lab stock

PLC5 Lab stock

Hep3B Lab stock

**Experimental Models: Organisms/Strains**

Nude mice Ensiweier

**Recombinant DNA**

pLKO.1-puro Xian et al., 2017

pMD2.G Xian et al., 2017

psPAX2 Xian et al., 2017

pcDNA3.1-LRP1 Prof. Joachim Herz, UTSW, USA

pcDNA3.1(+)-3×Flag Lab stock

pcDNA3.1-Flag-β chain This paper

pCS2-Flag-OGA Dr. Haishan Gao, Westlake University, PR China

pHAGE-CMV-MCS- IZsGreen Lab stock

pcDNA3.1-UFL1-HA Youbao Biotechnology Co., Ltd.,Changsha, China

pcDNA3.1-HA-ubiquitin Kamitani et al., 1997 Addgene (#18712)

**Software and Algorithms**

Graphpad Prism Graphpad Software https://www.graphpad.com

Image J Schneider et al., 2012 https://imagej.nih.gov/ij

**Table S3.** Sequence of Oligonucleotide.

**Name Sequence (5’-3’)**

**Primer sequences for knockdown**

shLRP1-1 Forward: CCGGCGCCGGATGTATAAATGTAAACTCGAGTTTAC ATTTATACATCCGGCGTTTTTG

Reverse: AATTCAAAAACGCCGGATGTATAAATGTAAACTCGA GTTTACATTTATACATCCGGCG

shLRP1-2 Forward: CCGGGGCAAGCGAGCACAGTATTATCTCGAGATAA TACTGTGCTCGCTTGCCTTTTTG

Reverse: AATTCAAAAAGGCAAGCGAGCACAGTATTATCTCGA GATAATACTGTGCTCGCTTGCC

shCtrl Forward: CCGGCCTAAGGTTAAGTCGCCCTCGCTCGAGCGA GGGCGACTTAACCTTAGGTTTTTG

Reverse: AATTCAAAAACCTAAGGTTAAGTCGCCCTCGCT CGAGCGAGGGCGACTTAACCTTAGG

**siRNA sequence (5’→3’)**

siCtrl TTCTCCGAACGTGTCACGTdTdT

siNF-κB p65 GCCCUAUCCCUUUACGUCAdTdT

siUFL1 CCTGTGCATTTAATCACTGAAdTdT

**Primer sequences for molecular cloning**

pcDNA3.1-α chain-Flag Forward: TGGCGGCCGCTCGAGGCCACCATGCTGACCCCG

Reverse: GAAGGGCCCTCTAGACTATCGCCGGTGGCGGTTGG

pcDNA3.1-β chain-Flag Forward: CCTCGAGCCGCCATGCAGATTGACCGGGGTGTCACC

Reverse: GTCTTTGTAGTCGAATGCCAAGGGGTCCCCTATCTCG

pcDNA3.1-β∆ chain-Flag Forward: ACCGAGCTCGGATCCGCCACCATGCCCCGGCCT

Reverse: CCACACTGGACTAGTTGCCAAGGGGTCCCCTATCT

Lentivirus-β∆ chain Forward: CTAGCTAGCGCCACCATGCTGACCCCGCCG

Reverse: GCTCTAGATCATGCCAAGGGGTCCCCTATCTC

**Primer sequence (5’→3’)**

*Ogt*-Forward TCCTGATTTGTACTGTGTTCGC

*Ogt*-reverse AAGCTACTGCAAAGTTCGGTT

*Oga*-Forward GAAGGAGAGTCAAGCGACGTT

*Oga*-reverse TCCATAACCCAAGGTCTTCCAT

*Gfpt1*-Forward GAGATGCCGGCGTTTGATTC

*Gfpt1*-reverse TCCACCATCACAGGCAACTC
